# Metabolomics Reveals Distinct Carbon and Nitrogen Metabolic Responses to Magnesium Deficiency in Leaves and Roots of Soybean [*Glycine max* (Linn.) Merr.]

**DOI:** 10.3389/fpls.2017.02091

**Published:** 2017-12-12

**Authors:** Na Yang, Jiali Jiang, Huiling Xie, Mengyan Bai, Qinzhen Xu, Xiaoguo Wang, Xiaomin Yu, Zhichang Chen, Yuefeng Guan

**Affiliations:** ^1^College of Resources and Environment, Fujian Provincial Key Laboratory of Haixia Applied Plant Systems Biology, Fujian Agriculture and Forestry University, Fuzhou, China; ^2^FAFU-UCR Joint Center, Fujian Provincial Key Laboratory of Haixia Applied Plant Systems Biology, Haixia Institute of Science and Technology, Fujian Agricultural and Forestry University, Fuzhou, China; ^3^College of Life Science, Fujian Agriculture and Forestry University, Fuzhou, China; ^4^Root Biology Center, Haixia Institute of Science and Technology, Fujian Agriculture and Forestry University, Fuzhou, China

**Keywords:** magnesium deficiency, carbon allocation, primary metabolism, metabolomic, GC-TOF, soybean

## Abstract

Magnesium (Mg) deficiency, a widespread yet overlooked problem in agriculture, has been reported to retard plant growth and development, through affecting key metabolic pathways. However, the metabolic responses of plant to Mg deficiency is still not fully understood. Here we report a metabolomic study to evaluate the metabolic responses to Mg deficiency in soybean leaves and roots. Hydroponic grown soybean were exposed to Mg starvation for 4 and 8 days, respectively. Metabolic changes in the first mature trifoliolate leaves and roots were quantified by conducting GC-TOF-MS based metabolomic analysis. Principal component analysis (PCA) showed that Mg deficient plants became distinguishable from controls at 4 days after stress (DAS) at metabolic level, and were clearly discriminated at 8 DAS. Mg deficiency could cause large metabolite alterations on carbon and nitrogen metabolism. At 8 DAS, carbon allocation from shoot to root is decreased by Mg deficiency. Remarkably, most amino acids (such as phenylalanine, asparagine, leucine, isoleucine, glycine, glutamine, and serine) showed pronounced accumulation in the leaves, while most organic acids (including pyruvic acid, citric acid, 2-keto-glutaric acid, succinic acid, fumaric acid, and malic acid) were significantly decreased in the roots. Our study shows that the carbon and nitrogen metabolic responses are distinct in leaves and roots under Mg deficiency.

## Introduction

Magnesium (Mg) is one of the core macronutrients required for plant growth. Mg occupies the central position in the chlorophyll structure, and thus is critical for solar harvesting in photosynthesis (Verbruggen and Hermans, 2013). Moreover, Mg acts as a cofactors for many enzymes, such as phosphatases, kinases, and ATPases (Verbruggen and Hermans, 2013). Physiological processes that are characterized to require Mg include photosynthetic carbon dioxide (CO_2_) fixation, carbon allocation, chlorophyll formation, protein synthesis, generation of reactive oxygen species (ROS), and alleviation of aluminum and lead toxicity (Shaul, 2002; Cakmak and Kirkby, 2008; Bose et al., 2013; Hermans et al., 2013; Kobayashi and Tanoi, 2015; Shen et al., 2016).

Mg is the 8th most abundant mineral element on earth (Maguire and Cowan, 2002). However, the concentration of plant-available Mg varies among different soil types, from 0.12 to 8.5 mM (Westermann, 1996; Gransee and Führs, 2013). Mg^2+^ cation binds weakly to negative charged soil colloids and root cell walls, and is high mobile in compare with other cations (Chen et al., 2017a). As a result, Mg is subject to leaching in considerable amounts, which depends on the water balance, acidity (H^+^ ions), and the concentration of other cations in the soil (Gransee and Führs, 2013; Chen et al., 2017a). In acidic soil that are highly saturated with cations, such as H^+^, Al^3+^, and Mn^2+^, Mg leaching is sever, particularly in high rainfall zones (Gransee and Führs, 2013; Chen et al., 2017a).

Despite the risk of Mg loss, fertilization of Mg has long been ignored in many agricultural practice (Cakmak and Yazici, 2010; Senbayram et al., 2015). Currently, Mg deficiency in agriculture is a widespread problem, affecting productivity, and quality of crops (Guo et al., 2015; Senbayram et al., 2015). A characteristic symptom of Mg deficiency is interveinal chlorosis due to the degradation of chlorophyll molecule (Cakmak and Yazici, 2010; Hermans et al., 2013; Chen et al., 2017a). Nevertheless, chlorosis appears to be a late response to Mg deficiency, whereas earlier response of plants to Mg deficiency is photoassimilates accumulation in source leaves and reduction in sink tissues (Gransee and Führs, 2013). Within days of Mg deficiency, sugar and starch are observed to be accumulated in source leaves, which is associated with the impairment of carbon allocation to sink tissues (Cakmak et al., 1994a,b; Hermans et al., 2004, 2005; Cakmak and Kirkby, 2008). The accumulation of carbohydrates in Mg-deficient source leaves occurs before any noticeable changes of shoot growth are observed (Cakmak et al., 1994a; Hermans et al., 2004). This indicates that the effect of Mg-deficiency on carbon allocation is specific to the process of source-to-sink translocation of sugar. The high susceptibility of carbon allocation to Mg deficiency may be related to phloem loading of sucrose in source leaves. Mg deficiency is considered to affect phloem loading of sucrose by decreasing Mg-ATP availability, leading to decreased H+-ATPase activity and reduced proton gradient which energizes active phloem loading (Brooker and Slayman, 1983; Cakmak et al., 1994a; Cakmak and Kirkby, 2008). Under Mg deficiency, free amino acids were also found to accumulate in source leaves, which was explained as a result of either inhibited protein synthesis or retarded phloem export of assimilates (Fischer et al., 1998). Moreover, reduced photosyntheticCO_2_ fixation, altered respiration, production of ROS, altered water use efficiency and salt tolerance have also been observed as Mg deficiency responses (Terry and Ulrich, 1974; Cakmak and Marschner, 1992; Tewari et al., 2006; Farhat et al., 2015; Trankner et al., 2016; Chen et al., 2017b; Li et al., 2017).

Since Mg is an essential component of many metabolic and signaling pathways, the effects of Mg deficiency on metabolic processes appear to be rapid and differentiated in source and sink tissues,. In *Arabidopsis thaliana*, Mg starvation induced distinct transcriptomic responses in root and leaves within 24 h of Mg removal (Hermans et al., 2010a). Short-term responses Mg deficiency include the perturbation of the circadian clock genes in roots and the activation of abscisic acid (ABA) signaling genes. After 1 week of Mg starvation, 114 and 2,991 genes were identified to be differentially regulated in root and shoot, with a high distribution in the “metabolism” category (Hermans et al., 2010b). For instance, the up-regulation genes were enriched in the ethylene biosynthetic pathway, and in the ROS detoxification (Hermans et al., 2010b).

Despite the progresses made, the early metabolic profile of plants in response to Mg deficiency remains elusive. Metabolomic analysis permits simultaneous monitoring of precursors, intermediates, and products of metabolic pathways. It is a discovery tool that can detect global metabolic responses to environment stimuli. Here we performed a metabolomic analysis with gas chromatography time-of-fly mass spectrometry (GC-TOF/MS), to study the metabolic responses of soybean [*Glycine max* (Linn.) Merr.] mature leaves and roots to Mg deficiency, before the outbreak of visual symptoms. We paid our attention to primary metabolites, such as sugars, amino acids, and organic acids. The results showed that Mg deficiency could cause massive and distinct metabolite alterations in leaves and roots, respectively.

## Materials and methods

### Plant material and growth conditions

A soybean cultivar, Yue Chun 03-3, was used in this study. Yue Chun 03-3 is commercialized in south China, where Mg leaching is a severe problem accompanying soil acidification. Wild-type soybean plants were grown in a growth chamber at the following conditions: light intensity of 400 μmol photons m^−2^ s^−1^, 13 h/26°C day and 11 h/24°C night regime, humidity 65%. Seeds were sterilized by chlorine gas, and germinated on sterilized and moisturized vermiculite. Four days after germination, seedlings were transferred to hydroponic culture in tanks as described (Li et al., 2012). The components of hydroponic culture are as the following: NH_4_NO_3_ 0.4 mmol, Ca(NO_3_)_2_ 1.2 mmol, KNO_3_ 1.5 mmol, (NH_4_)_2_SO_4_ 0.3 mmol, KH_2_PO_4_ 0.25 mmol, MnSO_4_ 0.5 μmol, ZnSO4 1.5 μmol, CuSO_4_ 0.5 μmol, (NH4)·MO_7_O_24_ 1.5 μmol, NaB_4_O_7_ 0.2 μmol, Fe·EDTA 0.04 mmol, K_2_SO_4_ 0.8 mmol, MgCl_2_ 0.5 mmol. The pH of nutrient solution was adjusted to 5.8 by 1 M KOH every 2 days, to avoid acidification of the solution. The nutrient solution was changed every week. After 10 days culture, when the first trifoliolate leaf is fully expanded and considered “source leaf,” soybean plants were transferred to Mg free or control solution at 10 a.m. of the day. For Mg free solution, MgCl_2_ was omitted from the media.

### Sample harvesting

Plant samples for biomass analysis were harvested at 10 days after stress (DAS), heat dried at 60°C for 72 h in a drying oven and weighed. For metabolomic and starch content analysis, leaves and roots were harvested for analysis at 4 and 8 DAS at 10 am of the day (2 h after lights). The whole first trifoliolate leaves were used for leaves sample. For roots, the whole root systems are big and difficult to handle in sample extraction. Therefore, primary roots of 10 cm from root tip were taken. All samples were put into liquid nitrogen immediately after removal. For each treatment, six biological replicates were analyzed. For each biological sample, leaves or roots from three individual plants were pooled. Therefore, a total of 18 plants were sampled for each treatment. Frozen samples were grounded with mortar and pestle into fine powder, lyophilized with Labconco Centrivap cold trap concentrator (Fisher Scientific Inc., USA), then separated into three technical replicates for metabolomic analysis.

### Mg and chlorophyll content analyses

For Mg content, both roots and shoots of soybean were dried at 75°C for 2 days in a drying oven and the dry weight was weighed. After digested with concentrated HNO_3_, the concentration of Mg was determined by Atomic Absorption Spectrometer (AAS, PerkinElmer, Shelton, USA). For chlorophyll content, 0.25 g fresh leaves were extracted with 80% acetone, then centrifuged at 4,000 rpm for 4 min. The supernatant was measured for optical density by spectrometer (V-1600, MAPADA Instruments Co., Ltd., Shanghai, China) at the wavelength of 663 and 645 nm, respectively. Chlorophyll content was calculated as described (Porra et al., 1989).

### Starch content analysis

Starch content was determined by anthrone-H_2_SO_4_ method modified from McCready et al. (1950). Two milligrams lyophilized leaves and 20 mg lyophilized root samples were incubated in 1 mL 80% ethanol at 80°C for 30 min. The remained pellet was resuspended in water and incubated at 95°C for 15 min, and starch was determined by spectrophotometer (UV-1780, SHIMADZU Co., Kyoto, Japan) at the wavelength of 620 nm.

### Sample extraction for GC-TOF

For metabolomic analysis, high purity solvents and reagents were purchased in order to avoid appearances of interfering MS peaks and high background. Sample extraction was performed as previously described (Fiehn, 2006; Fiehn et al., 2008). Briefly, 5 mg lyophilized samples were added with 1 mL prechilled and degassed extraction buffer of chloroform, methanol, and water (1:2.5:1 [v/v/v]), then 12 μL Arabitol (1 mg/mL) was added as inner standard. The sample was vortexed for 10 s and shaken with mixing plate for 6 min at 4°C. After centrifuge for 2 min at 14,000 rcf, 500 μL supernatant was transferred to a new tube, and then dried in the Labconco Centrivap cold trap concentrator (Fisher Scientific Inc., Waltham, MA, USA). For derivatization, 80 μL of methoxyamine solution (15 mg/mL in pyridine) was added to each sample, and shaken for 2 h at 37°C, 200 rpm. Then 80 μL MSTFA was added to each sample, and shaken for 30 min at 37°C, 200 rpm. One hundred fifty microliters sample reaction solutions was transferred to glass vials suitable for the GC-MS auto-sampler. Among every 5 tested samples, 1 quality control sample (mixture of all tested samples) were also injected.

### Data acquisition by GC-TOF-MS

GC-TOF-MS profiling was performed using a 1 μL injection by auto-sampler onto a capillary column (RESTEK Rxi®- 5sil MS) (30 m length, 0.25 mm inner diameter, and 0.25 μm film thickness) (RESTEK Co., Bellefonte, PA, USA), and an Agilent G6890B gas chromatograph with splitless injection and electronic pressure control (Agilent Co., Santa Clara, CA) mounted to a Pegasus HT time-of-flight mass spectrometer (LECO Co., Saint Joseph, MI, USA).

Helium was used as the carrier gas with the gas flow rate through the column was 2 mL/min. GC start at 80°C and 2 min isothermal, then ramp with 15°C/min up to 330°C and 6 min isothermal. The transmission line temperature was at 275°C. Electron impact ionization at 70 V was employed, with an ion source temperature of 250°C. The ion source filament energy was set to 70 eV. After 240 s solvent delay, filament was turned on and mass spectra were acquired in full scan mode at mass resolving power *R* = 600 from m/z 45 to 500 at 20 spectra per second and 1,460 V detector voltage without turning on the mass defect option. Recording ended after 1,200 s.

For Retention indexes (RIs) calculation, a mixture of internal retention index (RI) markers was prepared using fatty acid methyl esters of a mixture of C12, C15, C19, C22, C32, C36 linear chain length, and was injected to each chromatogram as standard additions.

### Data processing and metabolite identification

Chromatograms were manually assessed after mass spectral deconvolution (Chroma TOF, Software Version 4.5X, LECO, St Joseph, MI, USA). Baseline Offset = 1, Smoothin = Auto, Peak width = 5 s, signal-to-noise ratio = 10. Peak height of at least three mass fragments representing each analyte was normalized using internal standardization by Arabitol. RIs were calculated with Retention index methods of Chroma TOF software by normalization with internal RI markers. Features were characterized from the database using the software integrated LECO/Fiehn Metabolomics Library, and the corresponding metabolites were designated by software with threshold of similarity > 800. For the carbohydrates, organic acids and amino acids that are described in this study, metabolite standards were purchased from Sigma (Sigma-Aldrich Co., St. Louis, MO, USA) or Fluka (Fluka Chemical Co., Milwaukee WI, USA) and an internal library was constructed to confirm the metabolite identification. These metabolites were identified by comparing retention time and mass spectra manually to the standards.

### Data analysis

Prior to statistical analysis, the peak area of metabolites in each sample was first normalized according to internal standard (arabitol) among samples. Then the relative content of each metabolite in each sample was transformed by comparison to the internal standard. In our data analysis, an average value from the three technical replicates was calculated by Excel, which was used as the value of each biological replicate in statistical analysis.

Heatmap was produced with MetaboAnalyst server (Xia and Wishart, 2011; Xia et al., 2015). In one sample from 8 DAS roots, the concentration of all metabolites was unusually low (exceedingly dark blue color in compare with other samples), which should be caused by experimental error (e.g., bad injection) instead of variability in normal distribution. This sample was thus excluded from the data set (Figure S2).

For multivariant analysis, the excel file with normalized data of biological replicates was uploaded to MetaboAnalyst website, and the principal component analysis (PCA) function was performed. For univariant analysis, both student's *t*-test and fold change analysis were performed using MetaboAnalyst, respectively.

## Results

### Experimental design for Mg deficiency and metabolomic analysis

**Figure 1:**
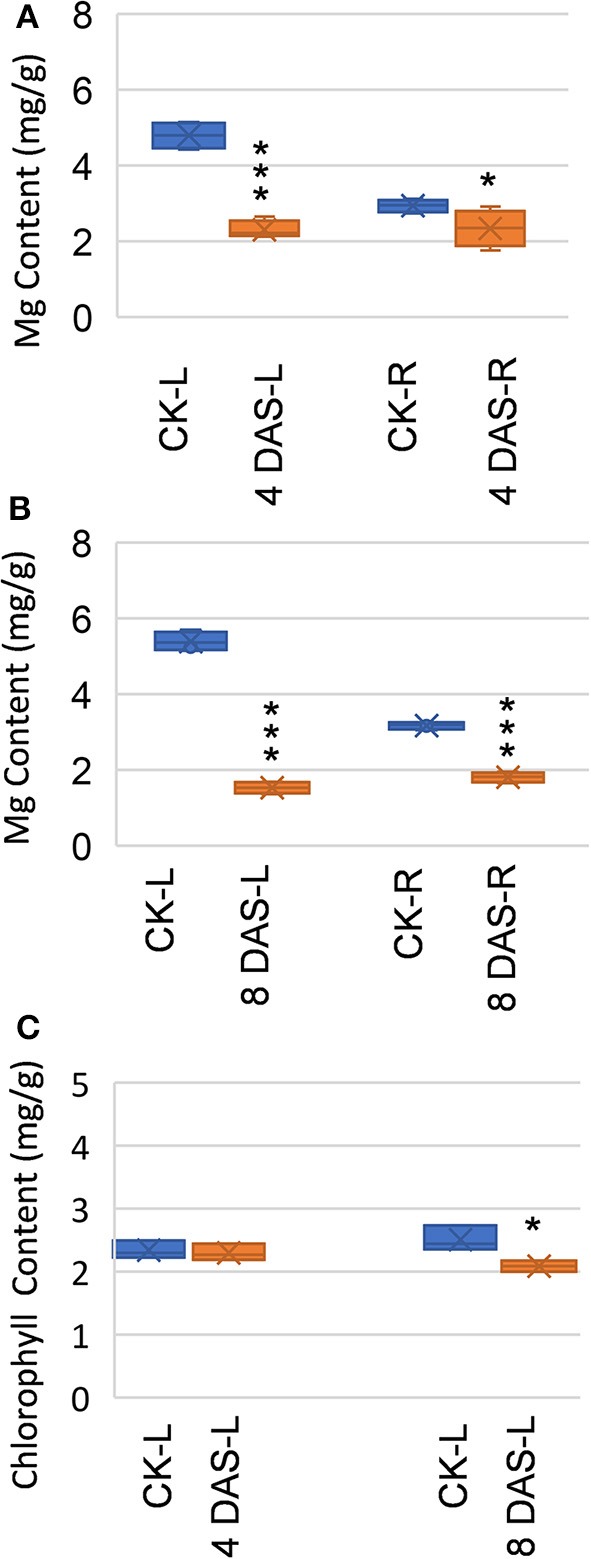
Effects of Mg deficiency on soybean plants. **(A,B)** Mg content of **(A)** 4 days after stress (DAS) and **(B)** 8 DAS soybean plants. **(C)** Chlorophyll content of 4 DAS and 8 DAS leaves. CK-L, control leaves at 4 or 8 days after stress; CK-R, control roots at 4 or 8 days after stress; 4 DAS-L, Mg deficient leaves at 4 days after stress; 4 DAS-R, Mg deficient roots at 4 days after stress; 8 DAS-L, Mg deficient leaves at 8 days after stress; 8 DAS-R, Mg deficient roots at 8 days after stress. ^***^p-value < 0.001 in *t*-test; ^*^p-value < 0.05.

We performed the Mg deficiency assays of soybean plants in a hydroponic system (Figure S1). Mg deficiency treatment was applied on 2-week-old plants (4 days on vermiculite followed by 10 days in hydroponic), when the first trifoliolate leaf is fully expanded and considered “source leaf.” To assess early metabolic responses which are direct consequences of Mg deficiency, we harvested first mature trifoliolate leaves and roots at 4 and 8 DAS, for metabolomic analysis with GC-TOF (Figure S1). At 4 DAS, Mg is already depleted in the mature leaves, while only moderately decreased in the roots (Figure 1A). This result indicated that Mg homeostasis is more sensitive in the mature leaves than in the roots. At this stage, no symptoms were observed on the stressed plants, and chlorophyll content is indistinguishable from normal plant (Figure 1C). Thus, the 4 DAS samples represent very early metabolic response when the stressed plants are still visually indistinguishable from control plants. At 8 DAS, Mg was depleted in both leaves and roots (Figure 1B), and chlorosis phenotypes start to appear on the younger leaves. In the 8 DAS mature trifoliate leaves, the chlorophyll content is only moderately decreased (*p*-value < 0.05) (Figure 1C). Therefore, the 8 DAS samples represents metabolic responses at the onset of Mg deficiency symptoms.

### Dramatic metabolic changes in Mg deficient soybean plants

**Figure 2:**
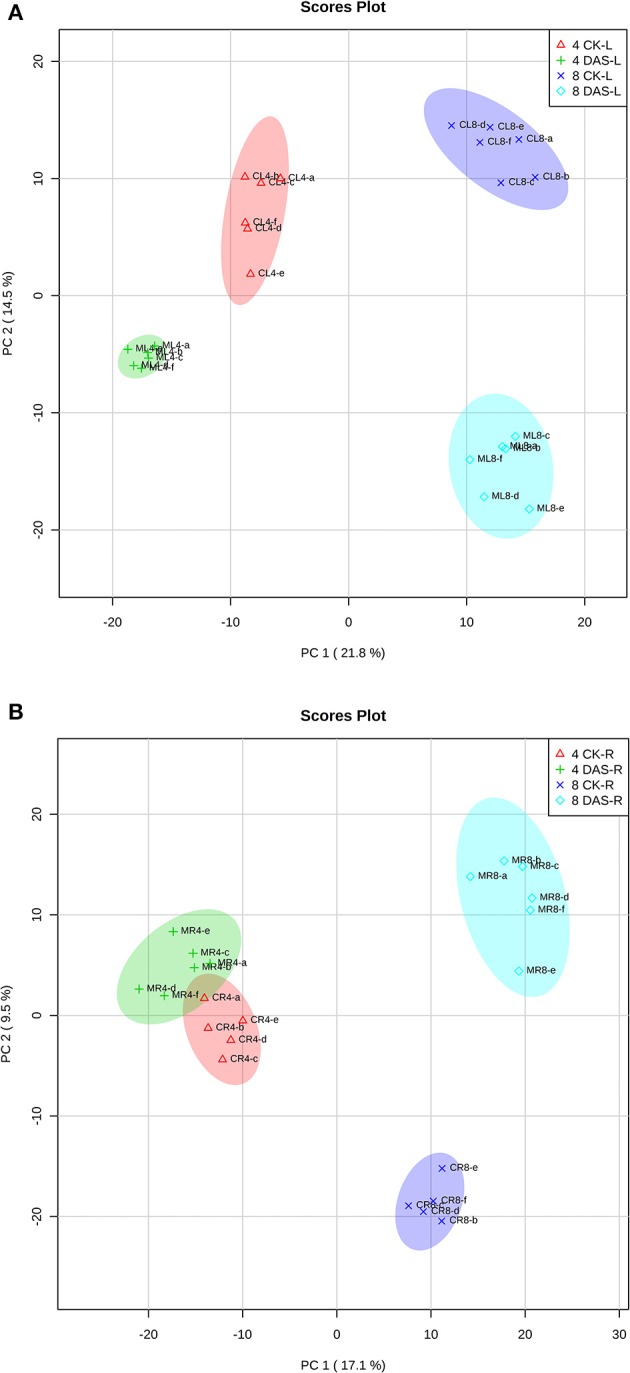
PCA analysis of metabolomic data. **(A)** Leaves samples **(B)** Roots samples. Four CK-L, control leaves at 4 DAS; 4 DAS-L, Mg deficient leaves at 4 DAS. Four CK-R, control roots at 4 DAS; 4 DAS-R, Mg deficient roots at 4 DAS. Eight CK-L, control leaves at 8 DAS; 8 DAS-L, Mg deficient leaves at 8 DAS. Eight CK-R, control roots at 8 DAS; 8 DAS-R, Mg deficient roots at 8 DAS.

After data normalization, PCA was performed to test for the presence of metabolic differences in responses to Mg deficiency, determine time point variation, and assess sample variation. To exclude the impact of steady-state tissue specificity that counts for the most differences among samples, we performed two PCA with leaves and roots samples, respectively. PCA revealed that two PC1 and PC2 principal components together accounted for 36.3 and 26.6% of the total variance within the leaves and roots datasets, respectively (Figures 2A,B). In both datasets, the first component, PC1, resolved the time points, which indicates a major difference between developmental stages. The second principal component, PC2, resolved the responses of Mg deficiency at the metabolic level. The PCA score plot showed that leaf samples from 4 DAS plants are distinguishable from the control, while the 4 DAS root samples were not clearly separated from the control (Figures 2A,B). This result is consistent with the more significant depletion of Mg in leaves than roots at 4 DAS. The differences between Mg deficiency and control plants are distinct at 8 DAS, which is consistent with the significant phenotype caused by Mg deficiency (Figures 2A,B).

For univariant analysis of differentially accumulated metabolites under Mg deficiency, a double filtering procedure with *t*-test and fold change was used. We set up a threshold of *p*-value < 0.05 in Student's *t*-test and more than 20% in fold change (|log2FC| > 0.26) for metabolites to be considered “responsive” (Misra et al., 2016; Zhu and Assmann, 2017). Furthermore, responsive features with *p*-value < 0.001 and fold change over 50% (|log2FC| > 0.59) were considered “very significant” changes (Table S1). A total of 85 identified metabolites showed statistically responses (|log2FC| > 0.26, *p*-value < 0.05) in at least one comparison between control and Mg deficient tissues (Table S1).

**Figure 3:**
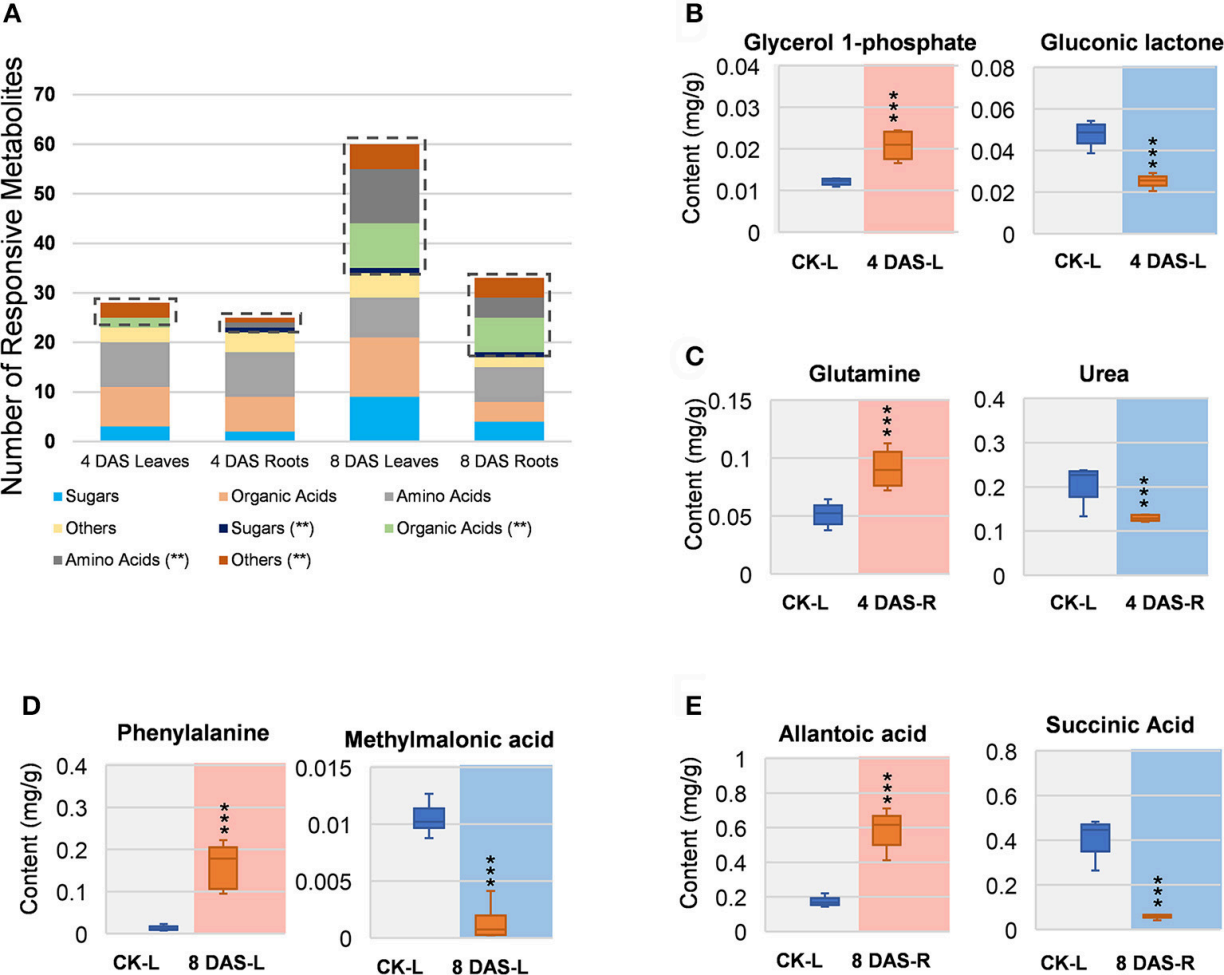
Univariant analysis of metabolomic data. **(A)** Summary of statistically changes metabolites in each sample. **(B–E)** Most increased and decreased metabolites in each sample. **(B)** 4 DAS leaves. CK-L, control leaves; 4 DAS-L, Mg deficient leaves at 4 DAS. **(C)** 4 DAS roots. CK-R, control roots; 4 DAS-R, Mg deficient roots at 4 DAS. **(D)** 8 DAS leaves. CK-L, control leaves; 8 DAS-L, Mg deficient leaves at 8 DAS. **(E)** CK-R, control roots; 8 DAS-R, Mg deficient roots at 8 DAS. ^***^p-value < 0.001 in *t*-test, |log2FC| > 0.59 in fold change.

In 4 DAS leaves, a total of 28 metabolites were altered by Mg deficiency, including 10 organic acids, 9 amino acids, 6 other metabolites, and 3 carbohydrates (Figure 3A). Only 5 metabolites were very significantly responsive, from which gluconic lactone was most depleted and D-glycerol-1-phosphate was the only significantly increased metabolite (Figure 3B, Table S1). In the roots, 25 metabolites were responsive to Mg deficiency at 4 DAS, including 7 organic acids, 10 amino acids, 5 other metabolites, and 3 carbohydrates (Figure 3A). Only 3 metabolites were very significantly changed including glutamine (Gln) that most induced and urea that was most decreased (Figure 3C, Table S1). A large metabolic variation was observed in 8 DAS leaves, with 60 metabolites identified to be responsive, including 21 organic acids, 19 amino acids, 10 carbohydrates, and 10 other metabolites (Figure 3A). Twenty-six metabolites were very significantly changed, with phenylalanine (Phe) showing most increase and methylmalonic acid showing most decrease (Figure 3D, Table S1). In 8 DAS roots, 33 metabolites were identified to be altered with 16 showing very significantly responses, which was less significant than 8 DAS leaves (Figure 3A). Allantoic acid was most induced among the increased metabolites, and succinic acid was most decreased in 8 DAS roots (Figure 3E, Table S1). The above results were consistent with the PCA analysis result that 8 DAS plants are more distinguishable from the control plants than 4 DAS.

### Carbon allocation of soybean is impaired in response to Mg deficiency

**Figure 4:**
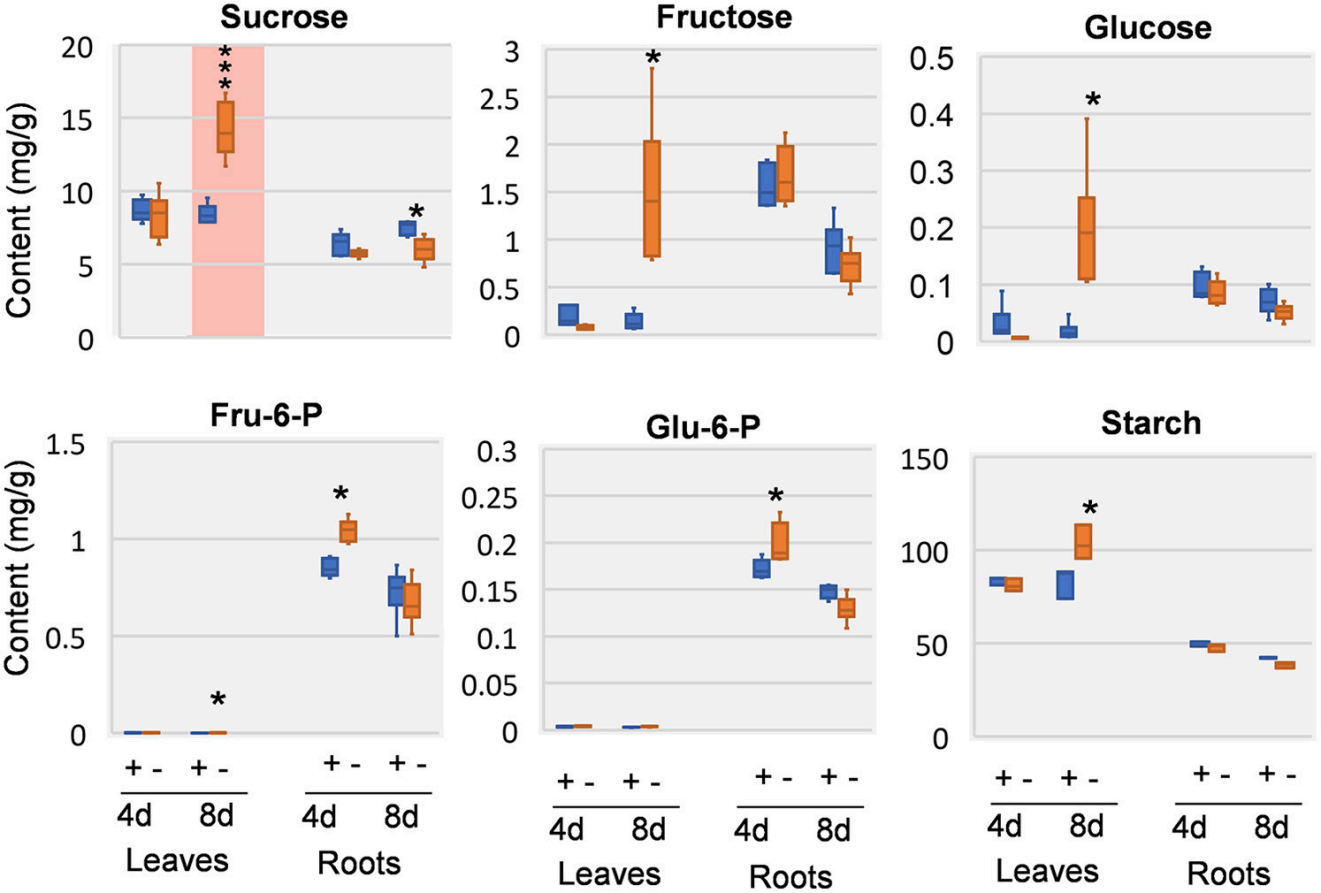
Carbohydrate changes in 8 DAS leaves and roots. + indicates control samples; – indicates Mg deficiency samples. 4 d, 4 DAS; 8 d, 8 DAS. Red background indicates very significant increase. ^***^p-value < 0.001 in *t*-test, |log2FC| > 0.59 in fold change; ^*^p-value < 0.05, |log2FC| > 0.26.

Previous study revealed that a characteristic phenotype of Mg starved plant is the impairment on carbon allocation (Cakmak et al., 1994a; Hermans et al., 2004, 2005; Cakmak and Kirkby, 2008). We therefore examined the effect of Mg deficiency on major carbohydrates involved in carbon allocation and respiration, including sucrose, glucose, fructose, glucose-6-phosphate (G6P), and fructose-6-phosphate (F6P). In the leaves, few responses was observed at 4 DAS, whereas highly increase of sucrose can be observed at 8 DAS. In addition, glucose, fructose, G6P, and F6P were also increased (Figure 4). In the roots, few significant changes were observed at either time point, except for sucrose was decreased at 8 DAS. Similar to sucrose, starch showed no changes in 4 DAS samples, and was accumulated in 8 DAS leaves and statistically decreased in 8 DAS roots (Figure 4).

Biomass allocation of soybean plants were analyzed at 12 days after stress (DAS). In compare with control plants, Mg starvation stressed plants showed reduced biomass in both shoots and roots (Figures S3A,B). Particularly, roots are more inhibited than shoots, resulting in a reduced root-shoot ratio (Figure S3C). This result confirmed that biomass allocation from shoot to root is affected by Mg deficiency in soybean plants, which is consistent with the impaired carbon allocation (Cakmak et al., 1994b; Hermans et al., 2005; Tewari et al., 2006; Cakmak and Kirkby, 2008; Cakmak and Yazici, 2010).

### Organic acids were significantly decreased in Mg deficient roots

**Figure 5:**
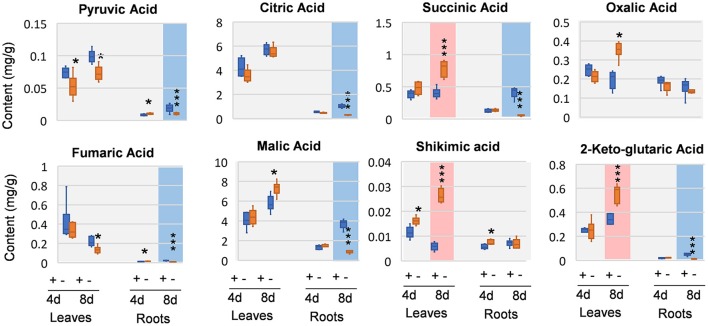
Organic changes in 8 DAS leaves and roots. + indicates control; – indicates Mg deficiency. 4 d, 4 DAS; 8 d, 8 DAS. Red background indicates very significant increase; blue background indicates very significant decrease. ^***^p-value < 0.001 in *t*-test, |log2FC| > 0.59 in fold change; ^*^p-value < 0.05, |log2FC| > 0.26.

We next analyzed organic acids that are intermediates in or closely related to glycolysis and tricarboxylic acid (TCA) cycle of respiration. In leaves, oxalic acid, and pyruvic acid was decreased at 4 DAS. In 8 DAS leaves, succinic acid, shikimic acid, and 2-keto-glutaric acid were very significantly increased while pyruvic acid and fumaric acid were decreased. In the roots, pyruvic acid and fumaric acid were increased, whereas citric acid and oxalic acid was deceased at 4 DAS. At 8 DAS, the metabolic responses of organic acids were pronouncedly decreased (Figure 5). Organic acids involved in glycolysis and TCA cycle, including pyruvic acid, citric acid, 2-keto-glutaric acid, succinic acid, fumaric acid, and malic acid were very significantly and strikingly decreased (Figure 5).

### Amino acids were significantly increased in Mg deficient leaves

**Figure 6:**
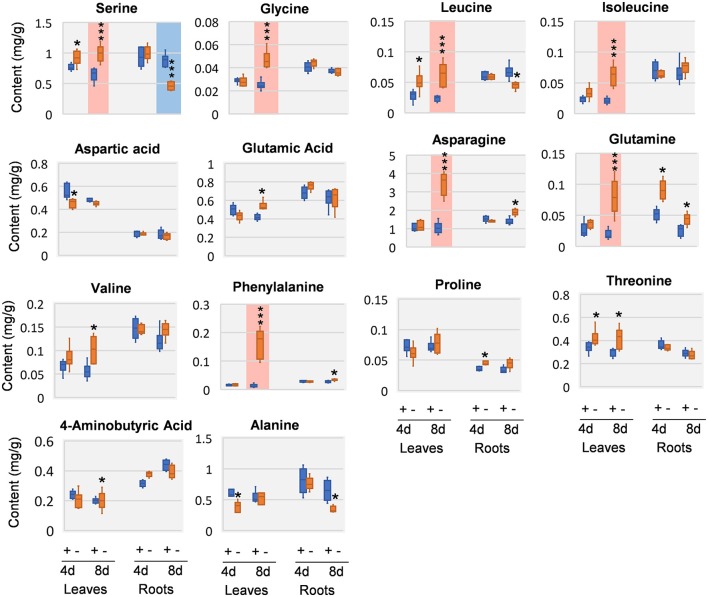
Amino acid changes in 8 DAS leaves and roots. + indicates control; – indicates Mg deficiency. 4 d, 4 DAS; 8 d, 8 DAS. Red background indicates very significant increase; blue background indicates very significant decrease. ^***^p-value < 0.001 in *t*-test, |log2FC| > 0.59 in fold change; ^*^p-value < 0.05, |log2FC| > 0.26.

Amino acids and close intermediates also exhibited dramatic responses to Mg deficiency. In compare with the little changes of carbohydrates and organic acids at 4 DAS, amino acids apparently were more responsive. In 4 DAS leaves, leucine (Leu) was significantly increased, while aspartic acid (Asp) and alanine (Ala) was decreased. In the roots, glutamic acid (Glu) was increased at 4 DAS. At 8 DAS, striking increase of most amino acids was observed in leaves, including Phe, asparagine (Asn), Leu, isoleucine (Ile), glycine (Gly), glutamine (Gln), and serine (Ser) (Figure 6). In contrast to the leaves, much fewer changes were observed in 8 DAS roots, with only Ala and Ser that were significantly decreased (Figure 6).

## Discussion

**Figure 7:**
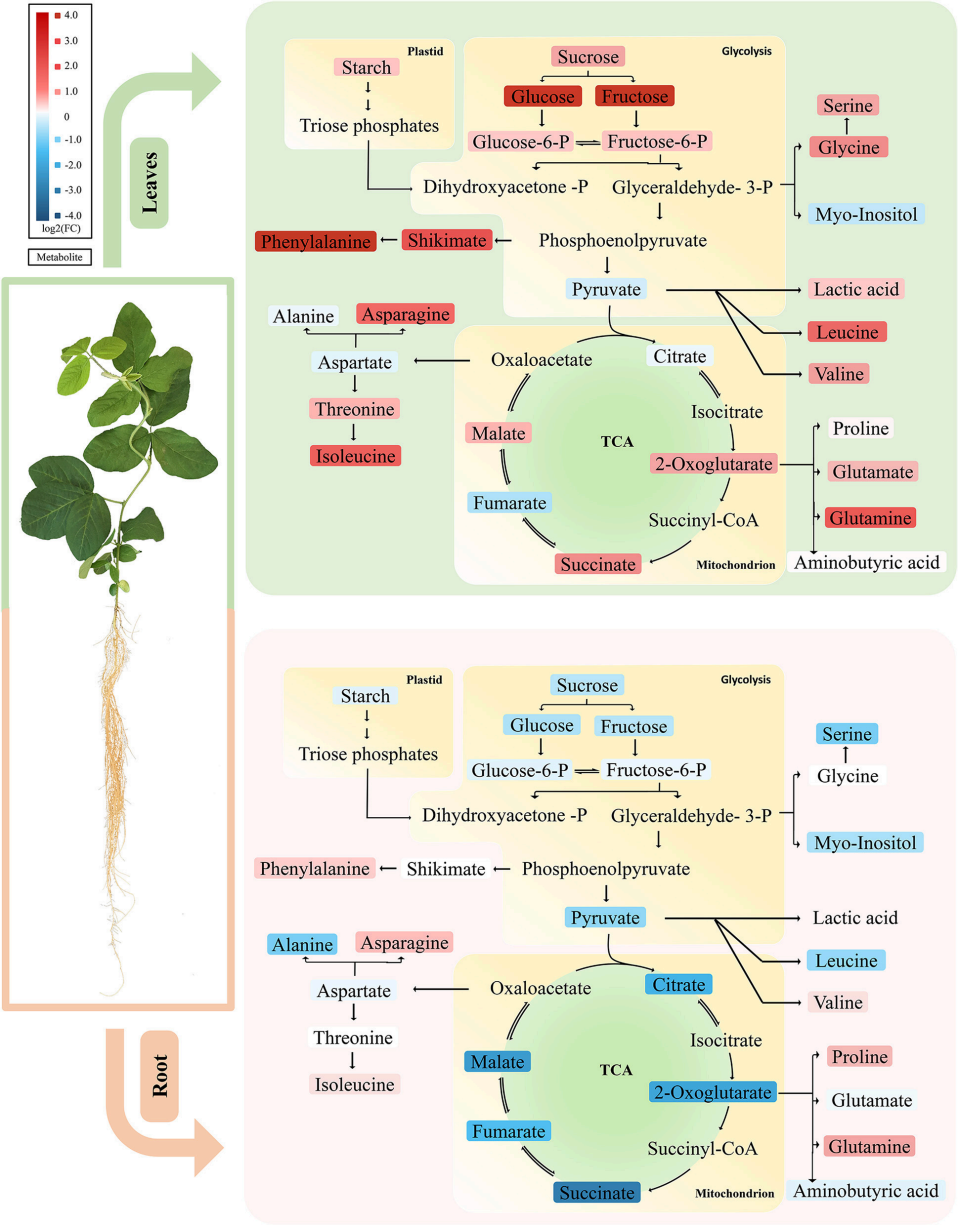
Primary metabolic pathways responses to Mg deficiency in leaves and roots. Upper panel represents metabolic responses in Mg deficient leaves, in which most carbohydrate and amino acids are increased. Lower panel showed metabolic responses in Mg deficient roots, in which most carbohydrates and organic acids tend to decrease. The up-regulated metabolites are marked in red and the down-regulated metabolites in blue. Tones of red or blue indicate the value of fold change. Upper panel, leaves; lower panel, roots.

This study provides an overview on the early metabolic phenotypes caused by Mg deficiency in mature leaves (source tissue) and roots (sink tissue). Metabolomic analysis allows simultaneous unbiased monitoring of precursors, intermediates, and products of metabolic pathways. In our metabolomic study with GC-TOF, we paid attention to primary metabolisms including sugars, glycolysis, TCA cycle, and amino acid biosynthesis. Multivariant and univariant analysis showed that the metabolism networks are affected at 4 DAS, while the effect was moderate with few metabolites showing significant changes. At 8 DAS, when the first mature trifoliate leaves are still visually indistinguishable from control, massive metabolism reprogramming was observed in both leaves and roots. Our results first support previous findings of the impairment of carbon allocation by Mg deficiency (Cakmak et al., 1994a; Hermans et al., 2004, 2005; Cakmak and Kirkby, 2008) (Figure 7). Moreover, the metabolic phenotype was distinct in leaves and roots at 8 DAS. Remarkably, most amino acids showed pronounced accumulation in the leaves, while most organic acids were significantly decreased in the roots (Figure 7).

### Carbohydrate allocation defects is more pronounced in source leaves at 8 DAS

In most plants, Mg deficiency leads to the marked increase in the shoot-to-root dry weight ratio, which is associated with sucrose and starch accumulation in leaves (Cakmak et al., 1994b; Hermans et al., 2005; Cakmak and Kirkby, 2008). So far, evidences support that the accumulation of sugar in Mg-deficient leaves is primarily due to impairment of sucrose export from the sources rather than to decreased sink activity (Cakmak and Yazici, 2010). In our study, we also found that the first trifoliolate leaves of soybean at 8 DAS exhibited significant increase of carbohydrates (starch, sucrose, glucose, and fructose) (Figure 7). Meanwhile, the roots showed only moderate decrease of sucrose and starch and no change in glucose and fructose (Figure 7). Therefore, our results indicate that the sugar unloading of roots at 8 DAS may not be severely affected yet, and the sugar accumulation in leaves is more likely an *in situ* consequence of Mg starvation.

### The root-specific depletion of organic acids may be attributed to the reprogramming of multiple metabolic pathways under Mg deficiency

It has been reported that external application of Mg facilitated release of organic acid anions from roots, to adapt acidic soil and Al-toxicity (Yang et al., 2007; Bose et al., 2013; Chen and Ma, 2013; Chen and Liao, 2016). However, the metabolic responses of organic acids to Mg deficiency in plants remains elusive. Our results showed that the 8 DAS soybean plants exhibit pronounced decrease of most organic acids specifically in the roots (Figure 7). Organic acids are intermediates of photosynthesis and respiration, and also serve as precursors for the synthesis of many other compounds (Drincovich et al., 2016). Due to the close metabolic relationship to primary and secondary metabolisms, organic acids are involved in the regulation of a broad range of basic cellular processes (Lopez-Bucio et al., 2000). Therefore, the mechanism underlying changes of organic acids under Mg deficiency are complicated and apparently a result of reprograming of multiple metabolic pathways.

Mg has been shown to be critical for activity of several enzymes in cellular respiration, a major carbon metabolic pathway affecting organic acid contents (Terry and Ulrich, 1974; Garfinkel and Garfinkel, 1985). The effects of Mg deficiency on respiration appeared to be temporally and spatially differentiated. In sugar beet leaves, rates of photorespiratory was significantly decreased. However, the respiration in the dark increased under Mg deficiency (Terry and Ulrich, 1974). In citrus, dark respiration was also increased in leaves, while decreased in the root (Li et al., 2017). In our study, it is possible that Mg deficiency might lead to inhibition of respiration which contributes to decrease of organic acids in 8 DAS roots. However, in 8 DAS leaves that were harvested during the day with presumably decreased respiration too, only fumaric acid was significantly decreased (Figure 7). Therefore, it seems that reduction in carbon metabolism may not be the only reason for organic acid depletion in 8 DAS roots.

A second factor that could be associated with organic acids depletion is the pH homeostasis. As abundant weak acids in plant cells, organic acids play an important role in maintenance of pH homeostasis. Mg has been shown to be required for H+-ATPase activities, a critical regulator of cytosolic pH (Brooker and Slayman, 1983; Yang et al., 2007). Therefore, the deficiency of Mg in the root may lead to inhibition of H^+^-ATPase, which would cause acidification of cytoplasm. When H^+^ pumping is inhibited, plant cells mainly employ organic acid metabolism for the cytoplasmic pH regulation (Lopez-Bucio et al., 2000). As a feedback response to Mg deficiency, organic acids biosynthesis may be decreased to balance the acidified cytosolic pH.

Lastly, ammonium assimilation has been documented to be closely related with organic acid metabolism. Organic acids are required for ammonium assimilation and amino acid synthesis (Goodchild and Givan, 1990; Hoffmann et al., 2007). When plants were grown on high ammonium, a depletion of organic acids was often observed, especially for malic acid and citric acid (Hachiya et al., 2012). In Mg deficient plant, ammonia uptake would increase due to reduced competition among positive-charged ions. In our study, urea is significantly accumulated in both leaves and roots of 8 DAS, which might be an indication of increased ammonium content (Figure 7). Given that primary ammonium assimilation takes place mostly in roots, the accumulation of ammonium may contribute to the depletion of organic acids in 8 DAS roots.

As introduced above, acidic soil with excess H^+^, Al, and Mn, is prone to Mg deficiency. Given that organic acids play an important role to alleviate Al and Mn toxicity (Rengel and Robinson, 1990; Senbayram et al., 2015), the Mg deficiency induced depletion of organic acids in roots may aggravates Al-toxicity in acid soil. Therefore, our results agree with previous opinions that external application of Mg at acidic soil condition is critical for protection of crops from Al and Mn toxicity (Yang et al., 2007; Chen and Ma, 2013).

### The leave-specific accumulation of amino acids under Mg deficiency may be due to N metabolism disruption

The induction of free amino acids under Mg deficiency has been documented by previous studies, but the detailed mechanism remains elusive (Fischer et al., 1998). Here we show that the accumulation of amino acids became very significant at relatively early stages (8 DAS) and was specific to the leaves. It was proposed that Mg deficiency may inhibits ribosome assembly and activity (Schuwirth et al., 2005), which is responsible for translation, and causes accumulation of free amino acids (Weiss et al., 1973; Sperrazza and Spremulli, 1983). Besides, the disruption of phloem loading may also lead to impaired nitrogen remobilization, and thus account for the accumulation of free amino acids in 8 DAS leaves. However, in Mg deficiency stressed spinach, although amino acids were also increased in source leaves, the amino acid concentration was not significantly affected in phloem sap Mg-deficient plants (Fischer et al., 1998). This result inferred that phloem remobilization of amino acids may not be limited by Mg deficiency.

Besides the above possibilities, we propose that chlorosis might contribute to the accumulation of amino acids (Fischer and Bremer, 1993; Fischer et al., 1998; Hermans et al., 2004, 2005). Chloroplast degradation, which may be due to self-imposed heterotrophic conditions in source leaf tissues, could lead to an enormous release of nitrogen in the form of amino acids (Sage et al., 1987; Crafts-Brandner et al., 1990; Masclaux-Daubresse et al., 2010; Araujo et al., 2011). In our study, amino acids strikingly increased in 8 DAS leaves, which coincide with the chloroplast degradation phenotype asparagine and glutamine, two key factors in nitrogen remobilization and long-range transport, were among the most increased amino acids in 8 DAS leaves (Figure 7) (Rochat and Boutin, 1991; Masclaux-Daubresse et al., 2010). Consistently, 2-oxoglutaric acid and succinic acid were also increased in 8 DAS leaves (Figure 7), which have been revealed to be alternative downstream products of amino acids from protein degradation to enter respiration (Araujo et al., 2011; Foyer et al., 2011). Therefore, we propose that chlorosis may be an important factor affecting free amino acid content in leaves under Mg deficiency.

## Conclusion

In summary, this study allowed us to monitor the Mg deficiency induced reprogramming of primary metabolism in soybean leaves and roots, which provides new insights into the impact of mineral nutrients on plant primary metabolisms. In general, the assimilates and metabolic intermediates tend to increase in source leaves (carbohydrates, amino acids), and decrease in roots (carbohydrates, organic acids) (Figure 7). These responses may be attributed to the reprogramming of multiple metabolic pathways under Mg deficiency. In future studies, other analytical methods, such as liquid chromatography–mass spectrometry, could be introduced to monitor secondary metabolism to complement the limitation of GC-TOF-MS method on primary metabolism. Moreover, genetic and molecular studies are required to answer elusive questions. For example, what are the key regulators controlling the disctinct metabolic responses of Mg deficiency in leaves and roots? What is the molecular mechanisms of retarded carbon allocation under Mg deficiency? What are the major factors leading to accumulated amino acids in the leaves and depleted organic acids in the roots under Mg deficiency?

## Author contributions

YG: contributed to the conception of the study, and wrote the manuscript. NY: performed most experiments, data analysis, and figure preparation. JJ, HX, MB, QX, and XW: helped in sample extraction, biomass measurement, Mg content measurement, and figure preparation. XY: help with GC-TOF and data analysis. ZC: contribute to the Mg and chlorophyll content measurement and manuscript preparation.

### Conflict of interest statement

The authors declare that the research was conducted in the absence of any commercial or financial relationships that could be construed as a potential conflict of interest.

## Supplementary material

Figure S1 Experimental design and sampling for metabolomic analysis. Soybean seeds were sterilized by chlorine gas, and germinated on sterilized and moisturized vermiculite. Four days after germination, seedlings were transferred to hydroponic culture in tanks for 10 days. When the first trifoliolate leaf is fully expanded and considered “source leaf,” soybean plants were transferred to Magnesium (Mg) free or control solution. For metabolomic and starch content analysis, leaves and roots were harvested for analysis at 4 and 8 days after stress (DAS). CK, control samples.

Figure S2 Heatmap and clustering of normalized data. Top 30 of the heatmap was shown. In this study, one sample from 8 DAS control roots was found to be outlier (green sample with dark blue plots in the right panel), as all metabolites are extraordinarily lower than other samples. This sample was removed from further analysis.

Figure S3 Biomass and root-shoot ratio after 12 days Mg deficiency. **(A)** Leave biomass. **(B)** Root biomass. **(C)** Root-shoot ratio. Blue background indicates very significant decrease. ^***^*p*-value < 0.001 in *t*-test.

Table S1 Responsive Metabolites in Mg Deficiency Treatment. Red color indicates statistical responses; Bold red color indicates very significant changes. Slashes indicate not detected in the samples.
