# Supplementary figures and images for: Metabolomics Reveals Distinct Carbon and Nitrogen Metabolic Responses to Magnesium Deficiency in Leaves and Roots of Soybean [Glycine max (Linn.) Merr.]
Source: Front Plant Sci. 2017 Dec 12;8:2091. doi: 10.3389/fpls.2017.02091 (PMC5733048; doi:10.3389/fpls.2017.02091)

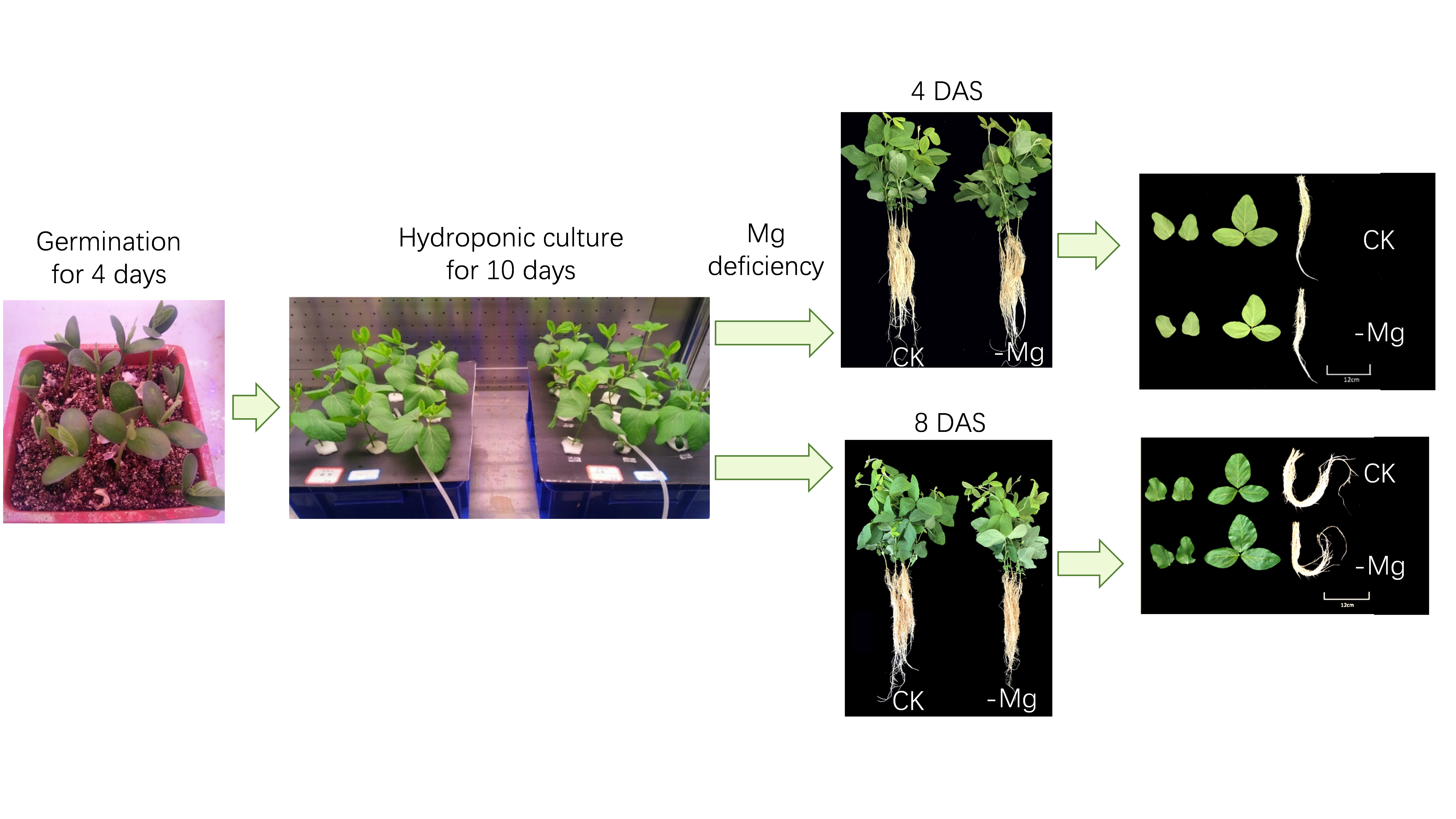

Supplement: Figure S1 — Experimental design and sampling for metabolomic analysis. Soybean seeds were sterilized by chlorine gas, and germinated on sterilized and moisturized vermiculite. Four days after germination, seedlings were transferred to hydroponic culture in tanks for 10 days. When the first trifoliolate leaf is fully expanded and considered “source leaf,” soybean plants were transferred to Magnesium (Mg) free or control solution. For metabolomic and starch content analysis, leaves and roots were harvested for analysis at 4 and 8 days after stress (DAS). CK, control samples. [file Image1.JPEG]

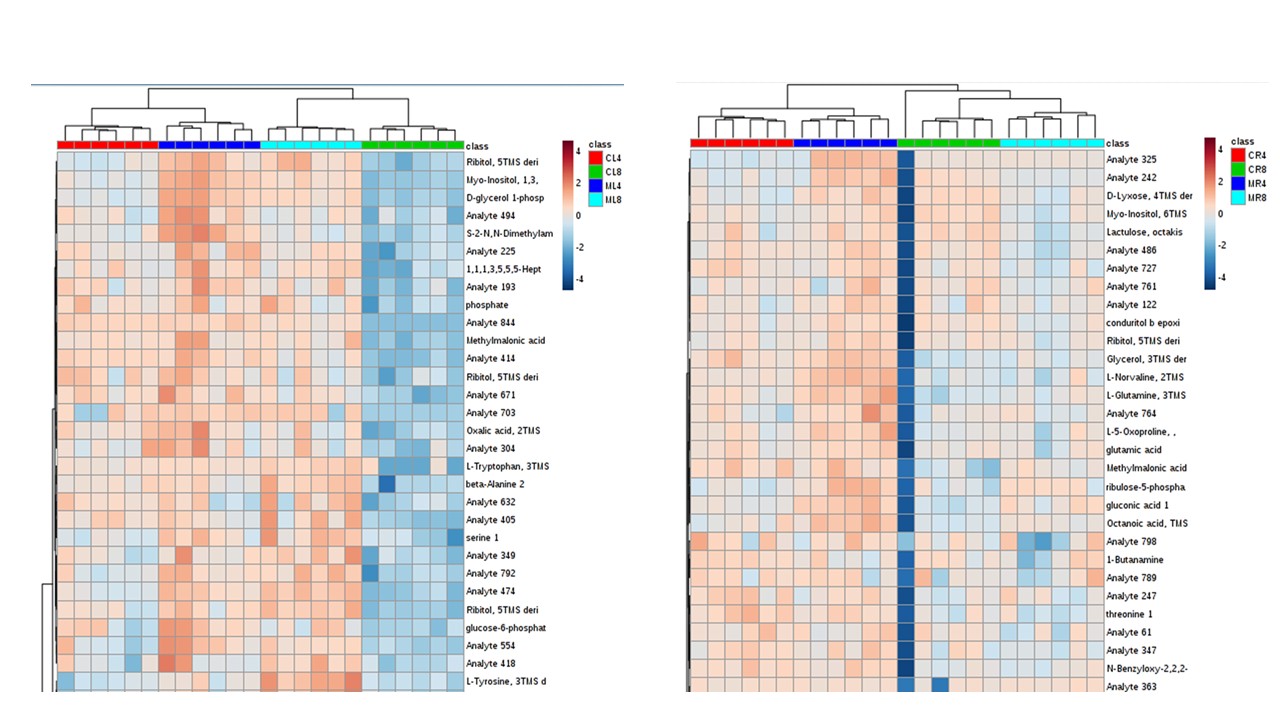

Supplement: Figure S2 — Heatmap and clustering of normalized data. Top 30 of the heatmap was shown. In this study, one sample from 8 DAS control roots was found to be outlier (green sample with dark blue plots in the right panel), as all metabolites are extraordinarily lower than other samples. This sample was removed from further analysis. [file Image2.JPEG]

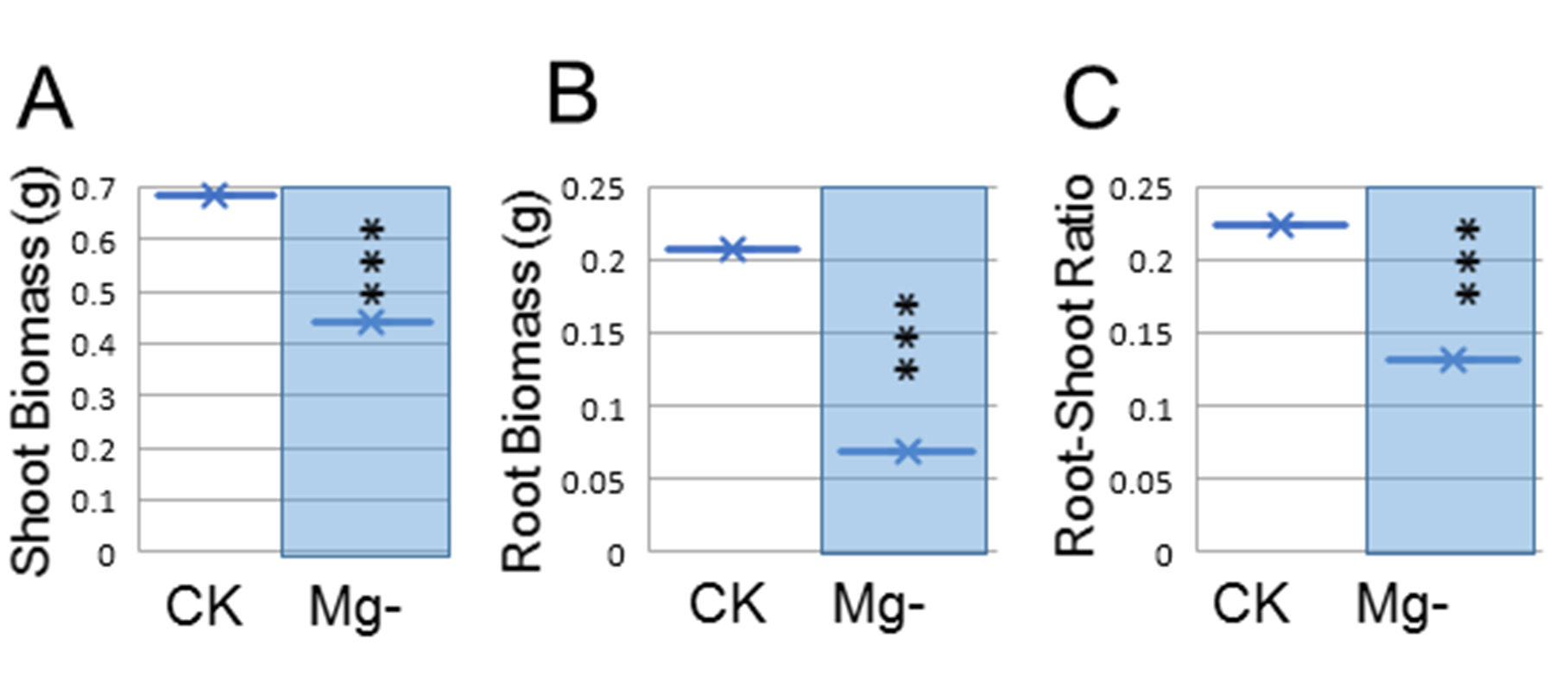

Supplement: Figure S3 — Biomass and root-shoot ratio after 12 days Mg deficiency. (A) Leave biomass. (B) Root biomass. (C) Root-shoot ratio. Blue background indicates very significant decrease. ***p-value < 0.001 in t-test. [file Image3.TIF]
