# Supplementary material for: Metabolomics Reveals Distinct Carbon and Nitrogen Metabolic Responses to Magnesium Deficiency in Leaves and Roots of Soybean [Glycine max (Linn.) Merr.]
Source: Front Plant Sci. 2017 Dec 12;8:2091. doi: 10.3389/fpls.2017.02091 (PMC5733048; doi:10.3389/fpls.2017.02091)
Supplement: Table S1 — Responsive Metabolites in Mg Deficiency Treatment. Red color indicates statistical responses; Bold red color indicates very significant changes. Slashes indicate not detected in the samples. [file Table1.DOCX]

|  | | | **4 DAS VS CK Leaves** | | **4 DAS VS CK Roots** | | **8 DAS VS CK Leaves** | | **8 DAS VS CK Roots** | |
| --- | --- | --- | --- | --- | --- | --- | --- | --- | --- | --- |
| **Metabolite** | **Retention time (seconds)** | **Mass** | log2(FC) | p-value | log2(FC) | p-value | log2(FC) | p-value | log2(FC) | p-value |
| **Sugars** |  | | | | | | | | | |
| Sucrose | 943.9 | 73 77 | -0.060 | 0.612 | -0.072 | 0.525 | **0.75** | **2.69****×10^-5^** | -0.34 | 2.68×10^-4^ |
| Glucose | 685.3  693.7 | 160 | -2.29 | 0.0541 | 0.12 | 0.749 | 3.44 | 1.71×10^-3^ | -0.43 | 0.164 |
| Fructose | 674.8  687.7 | 103 | -1.51 | 0.113 | 0.23 | 0.279 | 3.42 | 1.21×10^-3^ | -0.42 | 0.102 |
| G6P | 841.3 | 160 | 0.20 | 0.0662 | 0.24 | 1.41×10^-2^ | 0.31 | 1.91×10^-3^ | -0.21 | 0.0153 |
| F6P | 837.1 | 315 | 0.18 | 0.195 | 0.29 | 1.55×10^-4^ | 0.38 | 3.44×10^-4^ | -0.20 | 0.105 |
| Myo-Inositol | 762.4 | 73 | 0.30 | 8.31×10^-3^ | 0.27 | 0.0805 | -0.33 | 7.55×10^-6^ | **-0.65** | **5.28×10^-4^** |
| Xylose | 580.5  583.9 | 103 | -0.44 | 5.31×10^-3^ | 0.16 | 0.0695 | 0.33 | 0.0158 | -0.53 | 2.10×10^-4^ |
| Maltose | 976.9  980.9 | 204 | -0.10 | 0.241 | -0.080 | 0.400 | -0.26 | 5.66×10^-3^ | -0.30 | 0.0119 |
| Arabinose | 590.7 | 103 | -0.48 | 3.19×10^-2^ | 0.010 | 0.776 | 1.95 | 8.62×10^-3^ | 0.28 | 3.55×10^-4^ |
| Galactinol | 1052.1 1037.5 | 204 | -0.064 | 0.498 | **0.59** | **7.37×10^-4^** | -0.27 | 7.56×10^-3^ | -0.26 | 0.878 |
| ribulose-5-phosphate | 770 | 357 |  |  | 0.46 | 1.05×10^-3^ |  |  | 0.42 | 0.0731 |
| **Organic Acids** |  | | | | | | | | | |
| Succinic Acid | 408.3 | 147 | 0.31 | 0.0670 | 0.18 | 0.252 | **0.96** | **7.46×10^-5^** | **-2.82** | **5.18×10^-6^** |
| Pyruvic Acid | 248.7 | 73 | -0.48 | 0.0226 | 0.31 | 0.0161 | -0.44 | 1.64×10^-3^ | **-1.02** | **2.25****×10^-4^** |
| Malic Acid | 501.1 | 73 | 0.10 | 0.510 | 0.20 | 0.101 | 0.34 | 4.92×10^-3^ | **-2.12** | **3.45×10^-7^** |
| Fumaric Acid | 428.8 | 245 | -0.31 | 0.353 | 0.48 | 0.0171 | -0.70 | 7.66×10^-3^ | **-1.16** | **1.77×10^-5^** |
| Alpha-Ketoglutaric Acid | 543.7 | 73 | 0.021 | 0.921 | 0.31 | 0.0983 | **0.70** | **9.37×10^-5^** | **-2.04** | **2.29×10^-5^** |
| Citric Acid | 654.1 | 78 | -0.25 | 0.107 | -0.23 | 0.0123 | -0.058 | 0.396 | **-1.78** | **6.00×10^-7^** |
| Lactic acid | 255.3 | 147 | -0.47 | 1.65×10^-3^ | 0.096 | 0.622 | 0.32 | 6.96×10^-3^ | 0.15 | 0.437 |
| Shikimic acid | 649.1 | 204 | 0.49 | 1.94×10^-3^ | 0.43 | 8.08×10^-3^ | **2.17** | **3.32×10^-8^** | -0.15 | 0.436 |
| glycolic acid | 265.4 | 147 | **-0.71** | **2.37×10^-4^** | 0.19 | 5.19×10^-3^ | -0.40 | 1.81×10^-4^ | -0.072 | 0.363 |
| Oxalic acid | 481.6 | 73 | -0.078 | 0.462 | -0.23 | 0.126 | 0.55 | 1.61×10^-3^ | -0.13 | 0.604 |
| gluconic acid | 731.4 | 147 | -0.53 | 1.37×10^-3^ | 0.37 | 1.18×50^-3^ | 0.28 | 0.0238 | 0.51 | 0.0416 |
| beta-Mannosylglycerate | 909.8 | 204 | -0.41 | 3.88×10^-3^ | 0.077 | 0.854 | -0.46 | 6.41×10^-4^ | **-1.31** | **1.04×10^-6^** |
| Saccharic acid | 742.6 | 73 | -0.40 | 8.59×10^-4^ | 0.30 | 8.19×10^-5^ | -0.45 | 1.40×10^-5^ | 0.040 | 0.640 |
| Pipecolic acid | 437.4 | 156 | 0.53 | 0.209 | -0.061 | 0.869 | **2.46** | **1.78×10^-4^** | 0.78 | 0.0148 |
| Methylmalonic acid | 349.8 | 56 | -0.42 | 8.49×10^-3^ | -0.022 | 0.717 | **-3.13** | **1.92×10^-4^** | 0.36 | 0.0220 |
| Threonic acid | 535.3 | 73 | 0.15 | 0.599 | 0.41 | 9.12×10^-5^ | 0.20 | 0.0376 | -0.12 | 0.846 |
| Myristic acid | 818.4 668 | 117 | -0.12 | 0.0906 | 0.27 | 5.52×10^-3^ | -0.15 | 0.0869 | 0.29 | 0.107 |
| linoleic acid | 809.9 | 79 | -0.072 | 0.560 | 0.11 | 0.390 | -0.084 | 0.656 |  |  |
| Caffeic acid | 780.1 | 219 | **-0.78** | **3.3×10^-6^** |  |  | **-0.86** | **2.38×10^-5^** |  |  |
| 4-Coumaric acid | 705.8 | 293 | -0.32 | 1.74×10^-3^ |  |  | -0.42 | 1.39×10^-4^ |  |  |
| Aconitic Acid | 623.0 | 67 | 0.11 | 0.676 |  |  | **0.59** | **4.60×10^-4^** |  |  |
| Ferulic acid | 764.1 | 338 | -0.15 | 0.0653 |  |  | -0.46 | 9.99×10^-5^ |  |  |
| Linolenic acid | 824.8 | 95 | -0.23 | 0.0183 |  |  | -0.33 | 1.31×10^-3^ |  |  |
| Dehydroascorbic Acid | 677.0 | 157 | -0.39 | 0.0829 |  |  | **0.71** | **4.66×10^-5^** |  |  |
| cis-Coutaric acid | 1041.6 1069.9 | 219 | 0.43 | 0.0587 |  |  | **1.07** | **7.60×10^-6^** |  |  |
| **Amino Acids** |  | | | | | | | | | |
| Proline | 399.7 | 142 | -0.24 | 0.143 | 0.37 | 1.36×10^-3^ | 0.12 | 0.428 | 0.35 | 0.0576 |
| Phenylalanine | 569.8 | 218 | 0.13 | 0.430 | -0.041 | 0.621 | **3.63** | **2.81×10^-5^** | 0.28 | 0.014 |
| Aspartic acid | 468.4  517.1 | 160 232 | -0.28 | 9.30×10^-3^ | -0.080 | 0.432 | -0.11 | 0.0397 | -0.17 | 0.324 |
| Asparagine | 555.7  568.8  588.7 | 159  188  73 | 0.039 | 0.875 | -0.16 | 0.0543 | **1.72** | **6.50×10^-6^** | 0.42 | 1.39×10^-3^ |
| Leucine | 384.1 | 158 | 0.84 | 0.0137 | -0.068 | 0.429 | **1.43** | **5.84×10^-4^** | -0.56 | 4.34×10^-3^ |
| Isoleucine | 396.6 | 158 | 0.50 | 0.0535 | - 0.17 | 0.242 | **1.59** | **1.70×10^-4^** | 0.20 | 0.316 |
| Glycine | 404.3 | 174 | -0.056 | 0.553 | 0.16 | 0.0883 | **0.92** | **8.86×10^-5^** | -0.049 | 0.429 |
| Glutamic acid | 565.4 | 246 | -0.22 | 0.0357 | 0.19 | 0.0289 | 0.37 | 3.35×10^-4^ | -0.082 | 0.524 |
| Glutamine | 634.7 | 156 | 0.47 | 0.0935 | 1.01 | 9.12×10^-3^ | **2.07** | **4.03×10^-4^** | 0.62 | 0.0149 |
| Valine | 351.4 | 144 | 0.34 | 0.147 | -0.042 | 0.680 | 0.86 | 8.59×10^-3^ | 0.25 | 0.104 |
| Alanine | 282.5 | 116 | -0.67 | 2.31×10^-3^ | 0.093 | 0.723 | -0.036 | 0.800 | -0.96 | 2.30×10^-3^ |
| 4-Aminobutyric Acid | 523.1 | 174 | -0.22 | 0.197 | 0.29 | 4.52×10^-4^ | 0.0068 | 0.970 | -0.15 | 0.110 |
| Threonine | 447.2 | 73 | 0.30 | 0.0425 | -0.092 | 0.262 | 0.49 | 0.0107 | -0.080 | 0.471 |
| Serine | 433.3 | 73 | 0.26 | 0.0102 | 0.19 | 0.261 | **0.62** | **7.10×10^-4^** | **-0.94** | **4.75×10^-5^** |
| 3-Cyanoalanine | 439.6 | 141 | -0.61 | 1.17×10^-3^ | -0.23 | 0.109 | 0.66 | 0.0142 | 0.34 | 0.111 |
| L-homoserine | 481.2 | 128 | -1.82 | 0.0437 | 0.42 | 8.93×10^-3^ | -1.16 | 0.121 | -0.53 | 0.0539 |
| L-Tyrosine | 703.8 | 218 | 0.76 | 0.0213 | -0.26 | 0.229 | **2.28** | **3.10×10^-6^** | 0.76 | 0.0238 |
| L-Tryptophan | 808.2 | 202 | 0.54 | 0.0479 | -0.77 | 0.0228 | 2.69 | 3.45×10^-3^ | 0.87 | 0.0172 |
| beta-Alanine | 470.7 | 174 | 0.016 | 0.878 | -0.062 | 0.511 | **0.88** | **3.84×10^-4^** | **0.68** | **4.66×10^-5^** |
| Citrulline | 557.3 | 188 | 0.19 | 0.242 | -0.18 | 0.327 | **2.39** | **1.00×10^-6^** | 0.61 | 0.0352 |
| L-cysteine | 534.7 | 218 | -0.15 | 0.0838 | 0.31 | 8.97×10^-3^ | 0.46 | 1.47×10^-4^ | 0.29 | 0.0557 |
| L-5-Oxoproline | 518.7 | 156 | -0.21 | 0.0845 | 0.35 | 0.0121 | 0.32 | 6.12×10^-3^ | 0.21 | 0.371 |
| O-acetylserine | 451.6 | 73 | -0.068 | 0.717 | **0.58** | **7.35×10^-4^** | -0.50 | 8.71×10^-3^ | -0.10 | 0.561 |
| L-Norvaline | 365.1 | 144 |  |  | 0.46 | 1.13×10^-5^ |  |  | 0.18 | 0.416 |
| **Others** |  | | | | | | | | | |
| D-glycerol 1-phosphate | 628.08 | 299 | **0.77** | **3.27****×10^-5^** | 0.43 | 0.0126 | **0.99** | **1.00×10^-7^** | 0.24 | 0.281 |
| Gluconic lactone | 687.6 | 143 | **-0.92** | **4.10×10^-6^** | -0.034 | 0.611 | **-0.81** | **2.36×10^-5^** | 0.13 | 0.452 |
| Glycerol | 547.3 | 73 | 0.45 | 0.805 | -0.28 | 0.0884 | **2.43** | **3.92×10^-5^** | 0.38 | 0.166 |
| Methyl Phosphate | 327.3 | 241 | -0.56 | -0.381 | 0.20 | 0.0176 | -0.33 | 0.0397 | 0.054 | 0.582 |
| Zymosterol | 1118.1 | 129 | -0.51 | 5.27×10^-4^ | -0.11 | 0.389 | -0.055 | 0.559 | -0.18 | 0.581 |
| Stigmasterol | 1124.1 1139.7 | 83 129 | -0.38 | 4.91×10^-3^ | 0.035 | 0.914 | 0.13 | 0.226 | -0.20 | 0.0249 |
| phosphate | 383.8 | 299 | -0.81 | 0.203 | 0.75 | 0.0851 | 1.15 | 1.79×10^-3^ | **0.66** | **6.40×10^-6^** |
| 2,3-Butanediol | 442.7 493.3 | 117 | -0.25 | 0.197 | -0.0061 | 0.869 | -0.44 | 3.41×10^-3^ | -0.32 | 0.448 |
| Propylene glycol | 634.9 | 319 | -0.20 | 0.461 | -0.091 | 0.235 | 0.38 | 0.0302 | 0.31 | 0.147 |
| Trifluoroacetamide | 315.8 | 100 | -0.067 | 0.586 | 0.95 | 1.67×10^-3^ | 0.081 | 0.472 | **1.64** | **9.5×10^-6^** |
| Urea | 367.5 | 147 | -0.17 | 0.347 | 0.77 | 1.37×10^-3^ | **1.77** | **1.39×10^-6^** | **1.04** | **2.94×10^-5^** |
| Phytol | 793.4 | 143 | **-0.69** | **1.42×10^-4^** |  |  | **-0.59** | **1.13****×10^-5^** |  |  |
| Threitol | 509.9 | 103 | -0.30 | 4.24×10^-3^ |  |  | -0.30 | 7.11×10^-4^ |  |  |
| Neophytadiene | 665.1 | 68 | -0.18 | 0.0181 |  |  | **-0.66** | **6.00×10^-7^** |  |  |
| Putrescine | 620.4 | 174 |  |  | 0.60 | 1.92×10^-3^ |  |  | 0.92 | 9.34×10^-3^ |
| Allantoic acid | 758 | 100 |  |  | -0.43 | 0.0326 |  |  | **1.91** | **5.21×10^-5^** |
| Monoolein | 981 | 79 |  |  | 0.12 | 0.389 |  |  | -0.55 | 3.16×10^-3^ |
| 3-phosphoglycerate | 647.2 | 211 |  |  | **0.64** | **5.59×10^-4^** |  |  | **1.44** | **3.40×10^-5^** |
